# Piezoresistive temperature sensors fabricated by a surface micromachining CMOS MEMS process

**DOI:** 10.1038/s41598-018-35113-z

**Published:** 2018-11-20

**Authors:** Chunhua Cai, Junyan Tan, Di Hua, Ming Qin, Nianfang Zhu

**Affiliations:** 1 College of Internet of Things, Hohai University, Nanjing, 210098 China; 2 Jiangsu Key Laboratory of Power Transmission & Distribution Equipment Technology, Hohai University, Nanjing, 210098 China; 3 Key Laboratory of MEMS of Ministry of Education, Southeast University, Nanjing, 210096 China

## Abstract

This paper presents a micromachined monocrystalline silicon piezoresistive temperature sensor fabricated by a surface micromachining CMOS (Complementary Metal Oxide Semiconductor) MEMS (Micro-Electro-Mechanical System) process. The design of the temperature sensor is based on the structure of the multi-layer cantilever beam and the bimetallic effect. The temperature change of the cantilever beam is translated into the change of the piezoresistance’s value. The test results show that the sensitivities of the sensors are 27.9 mV/°C with 100 Ω/▯ piezoresistance between −40 °C to 60 °C and 7.4 mV/°C with 400 Ω/▯ piezoresistance between −90 °C to 60 °C. The temperature sensor proposed in this paper can be used in radiosondes for its low operating temperature (as low as −90 °C), small size (below 1 mm^2^) and low heat capacity.

## Introduction

With the development of micro-fabrication process and monolithic integration technology^1^, many types of miniature temperature sensors have been studied and applied widely. Compared with conventional temperature sensors, the merits of miniature temperature sensors are lower response time, more power-saving and lower cost^2,3^. It is well known that CMOS manufacturing process is a commercial method to fabricate Si-based miniature temperature sensors, which adopt the explicit temperature dependence of diodes, Si bipolar transistors or thermal resistances^4,5^.

As one of the most widely used temperature sensors, IC integrated temperature sensor based on PN junction has the advantages of simple structure, low cost and low power consumption. However, the PN junction temperature sensor cannot operate below −55 °C^6,7^. As a result, it cannot be used in radiosondes, which requires the temperature sensor to operate at temperature as low as −90 °C. Furthermore, some types of miniature temperature sensors are lately proposed for low temperature detection fields^8^. Even so, there is almost no miniature temperature sensor compatible with CMOS manufacturing process that can operate in the range between −90 °C and 60 °C.

In order to produce a temperature sensor applicable in radiosondes, Dr. Ma in our lab proposed a micromachined monocrystalline silicon capacitive temperature sensor^8^. The experimental results show that the sensor provides a sensitivity of 7 fF/°C in the −70 to 100 °C range. However, the fabrication process needed wet etching on the backside of silicon substrate, which could lead to ion contamination on CMOS IC and is not compatible with CMOS process. In addition, this fabrication process used by Dr. Ma chose the SOI wafer as the substrate material^8^, which was expensive and increased the cost of the sensor.

**Figure 1:**
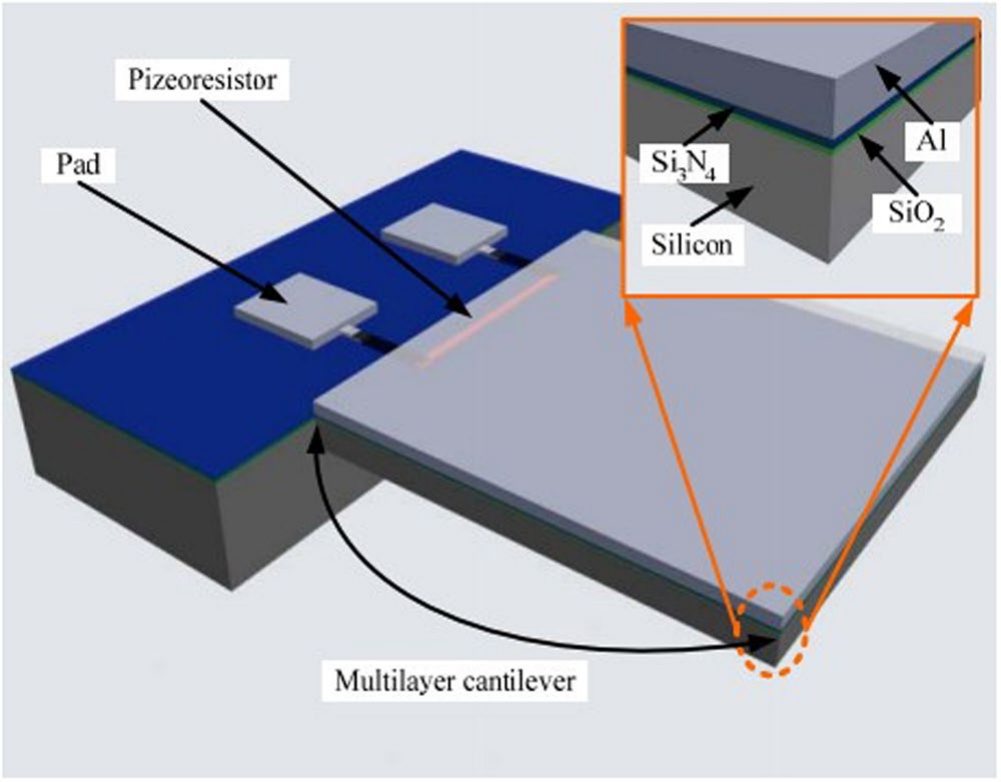
Multi-layer cantilever structure of the piezoresistive temperature sensor.

In this paper, a modified micromachined monocrystalline silicon piezoresistive temperature sensor is proposed to achieve miniaturization, high adaptability and low response time. As shown in Fig. 1, this temperature sensor is constructed as four multilayer cantilevers and fabricated by a surface monocrystalline silicon CMOS MEMS process. The bottom layer and the top layer of the multi-layer cantilever are monocrystalline silicon and Al, respectively. The Si_3_N_4_/SiO_2_ composite layer is between the silicon and Al. The silicon layer forms a piezoresistive element by the way of ion implantation.

The temperature sensors are fabricated by using the 6-inch production lines-CSMC Technologies, which is the first open 6-inch foundry in China and compatible with the CMOS IC process. The piezoresistance of the temperature sensor is formed by ion implantation based on CMOS IC process. The thickness of the Si_3_N_4_/SiO_2_ composite layer cannot be modified due to the restrictions of the design rules of the OEM factory (CSMC Technologies). We could modify the thickness of the bottom layer (silicon) and the top layer (Al), and the length/width ratio of the multi-layer cantilever beam. The parameters of the MEMS multi-layer cantilever beam are shown in Table 1.

**Table 1:** The parameters of MEMS multi-layer cantilever beam.

Material	Young’s modulus (GPa)	Thermal expansion coefficient (10^−6^/°C)	Poisson’s ratio	Thickness (*μm*)	Length/Width
Al	70	25	0.33	0.5–5 *μm*	300–500 *μm*/500 *μm*
Si_3_N_4_	385	0.8	0.28	0.1 *μm*	300–500 *μm*/500 *μm*
SiO_2_	73	0.55	0.17	0.3 *μm*	300–500 *μm*/500 *μm*
Si	165	2.33	0.33	3–20 *μm*	300–500 *μm*/500 *μm*

## Design of the Piezoresistive Temperature Sensor

**Figure 2:**
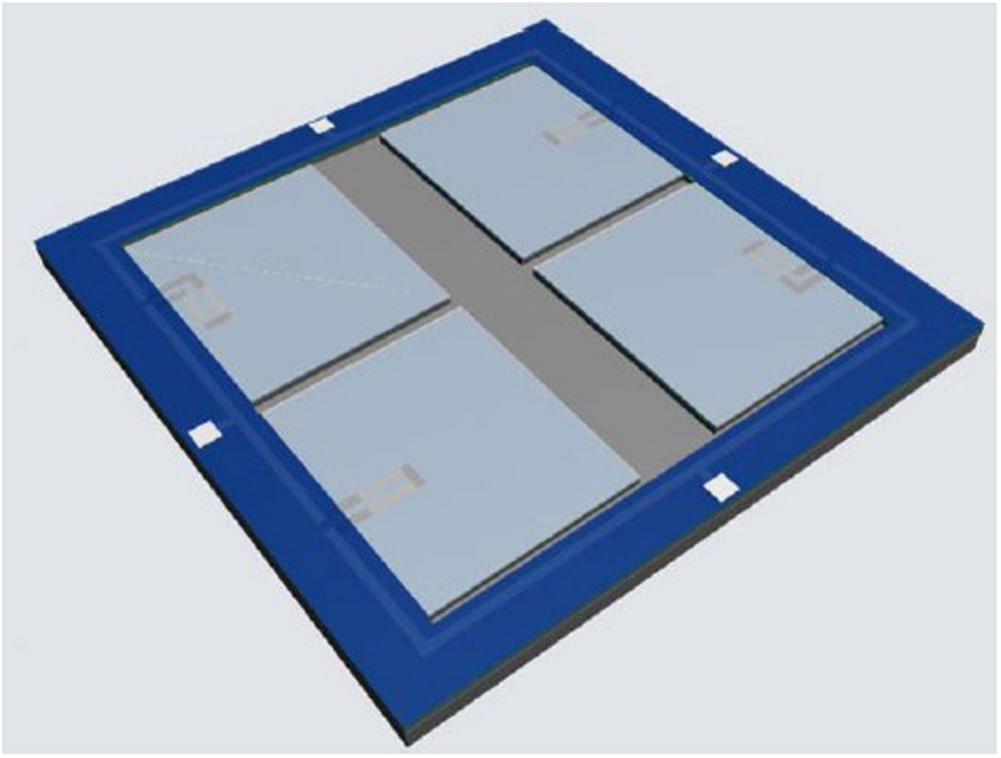
Illustration of multi-cantilever piezoresistive temperature sensor.

The thermal response of the piezoresistive temperature sensor is due to the different thermal expansion coefficients of silicon, Si_3_N_4_/SiO_2_ and Al materials. Due to the mismatches of the thermal expansion coefficient between the materials within the multilayer structure, thermal stress is generated when temperature varies. Under the stresses, the cantilever is bent upward or downward, as temperature decreases or increases. Due to the piezoresistive effect, the strain affects the resistivity of the piezoresistors, and the temperature variations are sensed and translated to the resistance changes. The piezoresistive effect is related to the direction of stresses and current: the changes of piezoresistances are usually opposite, when the direction of stresses is parallel or perpendicular to the direction of the current. The designed temperature sensor contains four piezoresistive elements, which are connected together according to Wheastone bridge, as shown in Fig. 2.

## Modeling of the Temperature Sensor

**Figure 3:**
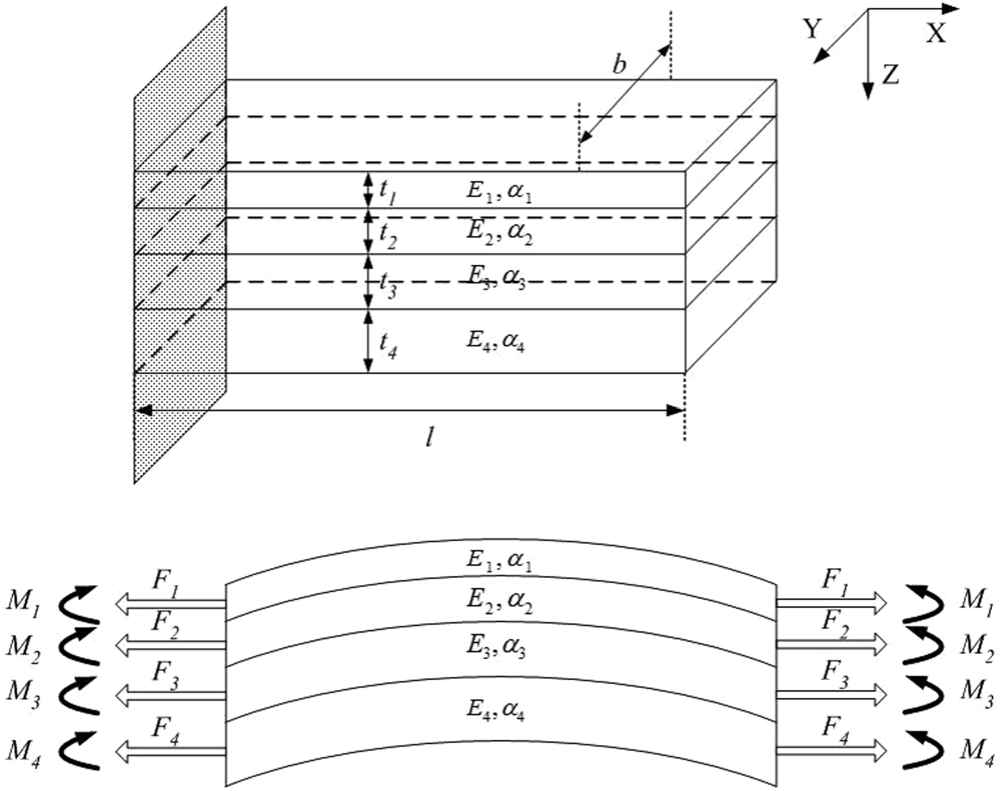
The model of the thermo-mechanical analysis.

Based on Timoshenko’s assumptions^9^, the multilayer cantilever thermal - mechanical response is analyzed^8,10^. Figure 3 shows the model of the thermo-mechanical analysis.

According to the model, axial tensile force *F*_*i*_ and bending moment *M*_*i*_ (*i* = 1, 2, 3, 4) act on the cross-section of a segment^11^. Respectively, *α*_*i*_ is the thermal expansion coefficient of the *i*^*th*^ layer, and *E*_*i*_ is the Young’s modulus of the *i*^*th*^ layer. The width of each layer is *b*, and *t*_*i*_ represents the thickness of the *i*^*th*^ layer.

Timoshenko has the following implicit assumptions:

- The material of each layer is homogeneous and has good elasticity.
- Uniformity of temperature across the beam.
- Physical parameters, such as the Young’s modulus and the thermal expansion coefficient, are regarded as constants.
- The direct stresses are always along the length of the cantilever beam.
- The strain at the interface between two adjacent materials is continuous.

Based on the above assumptions, the axial forces at any cross section shown in Fig. 3 must sum to zero.

1 $$\sum _{i=1}^{4}\,{F}_{i}={F}_{1}+{F}_{2}+{F}_{3}+{F}_{4}=0$$

The radii of curvature of each layer are approximately equal.

2 $${\rho }_{1}={\rho }_{2}={\rho }_{3}={\rho }_{4}=\rho $$

Due to the torque balance, it can be obtained:

3 $${F}_{1}(\frac{{t}_{1}}{2})+{F}_{2}({t}_{1}+\frac{{t}_{2}}{2})+{F}_{3}({t}_{1}+{t}_{2}+\frac{{t}_{3}}{2})+{F}_{4}({t}_{1}+{t}_{2}+{t}_{3}+\frac{{t}_{4}}{2})={M}_{1}+{M}_{2}+{M}_{3}+{M}_{4}$$

where axial moment *M*_*i*_ = *E*_*i*_*I*_*i*_/*ρ*. Based on equation (3), the beam equilibrium in terms of axial forces and curvature can be shown as:

4 $${F}_{1}(\frac{{t}_{1}}{2})+{F}_{2}({t}_{1}+\frac{{t}_{2}}{2})+{F}_{3}({t}_{1}+{t}_{2}+\frac{{t}_{3}}{2})+{F}_{4}({t}_{1}+{t}_{2}+{t}_{3}+\frac{{t}_{4}}{2})=\frac{1}{\rho }({E}_{1}{I}_{1}+{E}_{2}{I}_{2}+{E}_{3}{I}_{3}+{E}_{4}{I}_{4})$$

The inertia moment is shown as:

5 $${I}_{i}=b{t}_{i}^{3}\mathrm{/12(}i=\mathrm{1,}\,\mathrm{2,}\,\mathrm{3,}\,\mathrm{4)}$$

Based on the continuity of strain at the interface between two adjacent materials, the following equations can be obtained:

6 $${\alpha }_{1}{\rm{\Delta }}T+\frac{{F}_{1}}{{E}_{1}{t}_{1}b}-\frac{{t}_{1}}{2\rho }={\alpha }_{2}{\rm{\Delta }}T+\frac{{F}_{2}}{{E}_{2}{t}_{2}b}+\frac{{t}_{2}}{2\rho }$$

7 $${\alpha }_{2}{\rm{\Delta }}T+\frac{{F}_{2}}{{E}_{2}{t}_{2}b}-\frac{{t}_{2}}{2\rho }={\alpha }_{3}{\rm{\Delta }}T+\frac{{F}_{3}}{{E}_{3}{t}_{3}b}+\frac{{t}_{3}}{2\rho }$$

8 $${\alpha }_{3}{\rm{\Delta }}T+\frac{{F}_{3}}{{E}_{3}{t}_{3}b}-\frac{{t}_{3}}{2\rho }={\alpha }_{4}{\rm{\Delta }}T+\frac{{F}_{4}}{{E}_{4}{t}_{4}b}+\frac{{t}_{4}}{2\rho }$$

9 $$\frac{{F}_{1}}{b}+\frac{{F}_{2}}{b}+\frac{{F}_{3}}{b}+\frac{{F}_{4}}{b}=0$$

We define the matrices A, B, C, D and F:

10 $$[\begin{array}{cccc}\frac{1}{{E}_{1}{t}_{1}} & -\,\frac{1}{{E}_{2}{t}_{2}} & 0 & 0\\ 0 & \frac{1}{{E}_{2}{t}_{2}} & -\,\frac{1}{{E}_{3}{t}_{3}} & 0\\ 0 & 0 & \frac{1}{{E}_{3}{t}_{3}} & -\,\frac{1}{{E}_{4}{t}_{4}}\\ 1 & 1 & 1 & 1\end{array}]=A$$

11 $$[\begin{array}{c}{t}_{1}+{t}_{2}\\ {t}_{2}+{t}_{3}\\ {t}_{3}+{t}_{4}\\ 0\end{array}]=B$$

12 $$[\begin{array}{c}{\alpha }_{1}-{\alpha }_{2}\\ {\alpha }_{2}-{\alpha }_{3}\\ {\alpha }_{3}-{\alpha }_{4}\\ 0\end{array}]=C$$

13 $${(\sum _{i\mathrm{=1}}^{4}{E}_{i}{I}_{i})}^{-1}[\begin{array}{cccc}\frac{{t}_{1}}{2} & {t}_{1}+\frac{{t}_{2}}{2} & {t}_{1}+{t}_{2}+\frac{{t}_{3}}{2} & {t}_{1}+{t}_{2}+{t}_{3}+\frac{{t}_{4}}{2}\end{array}]=D$$

14 $$[\begin{array}{c}{F}_{1}\\ {F}_{2}\\ {F}_{3}\\ {F}_{4}\end{array}]=F$$

So the equations (6), (7), (8), and (4) can be rewritten as:

15 $$\frac{AF}{b}-\frac{1}{2\rho }B+{\rm{\Delta }}TC=0$$

16 $$\frac{1}{\rho }=\frac{DF}{b}$$

Based on equations (15) and (16), the axial forces and the radius of curvature can be expressed as:

17 $$F=b{\rm{\Delta }}T(\frac{D{A}^{-1}C{A}^{-1}B}{D{A}^{-1}B-2}-{A}^{-1}C)$$

18 $$\frac{1}{\rho }=2{\rm{\Delta }}T\frac{D{A}^{-1}C}{D{A}^{-1}B-2}$$

For the further analysis of the thermal-stress response, it is assumed that:

19 $$J=2\frac{D{A}^{-1}C}{D{A}^{-1}B-2}$$

20 $$K=[\begin{array}{c}{K}_{1}\\ {K}_{2}\\ {K}_{3}\\ {K}_{4}\end{array}]=\frac{D{A}^{-1}C{A}^{-1}B}{D{A}^{-1}B-2}-{A}^{-1}C$$

**Figure 4:**
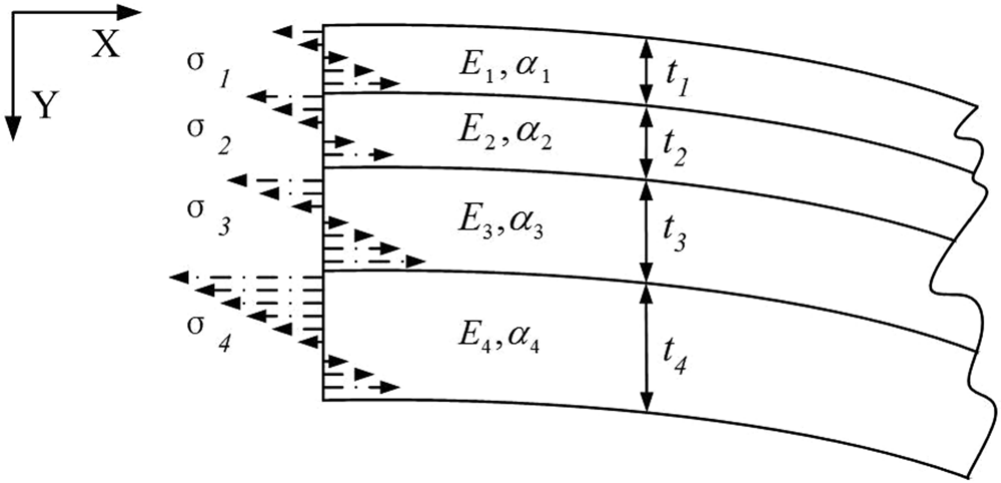
The model of the thermo-stress analysis.

As shown in Fig. 4 the stress *σ*_*i*_ in each layer can be calculated as:

21 $${\sigma }_{i}(h)=\frac{{F}_{i}}{b{t}_{i}}+\frac{{E}_{i}}{\rho }(\frac{{t}_{i}}{2}-h)=\frac{{K}_{i}{\rm{\Delta }}T}{{t}_{i}}+J{E}_{i}{\rm{\Delta }}T(\frac{{t}_{i}}{2}-h)$$

where *h* is the distance from any point to the top surface of each layer.

Based on equation (21), the stress is biggest on the top surface of each layer. The maximum stress on Si layer can be shown as:

22 $${\sigma }_{4max1=\frac{{K}_{4}{\rm{\Delta }}T}{{t}_{4}}+\frac{J{E}_{4}{\rm{\Delta }}T{t}_{4}}{2}}$$

## Output of the Temperature Sensor

Tufte O. N. and Stelzer E. L. had measured values of the three piezoresistance coefficients (*π*_11_, *π*_12_ and *π*_44_) and also obtained the temperature dependence of the piezoresistance coefficients of silicon layers having surface impurity concentration values from 10^18^ to 10^21^ cm^−3^ ^12^. In p-type silicon layers, the temperature dependence of the piezoresistance coefficient *π*_44_ decreased with increasing impurity concentration^12^.

In order to minimize the temperature dependence of the *π*_44_ coefficient, piezoresistances chosen in this paper are p-type and have high impurity concentration. The substrate is (100) n-type epitaxial silicon. The factory (CSMC Technologies) provided two types of piezoresistances with high impurity concentration. The square resistance of two piezoresistances are 100 Ω/▯ and 400 Ω/▯ respectively.

**Figure 5:**
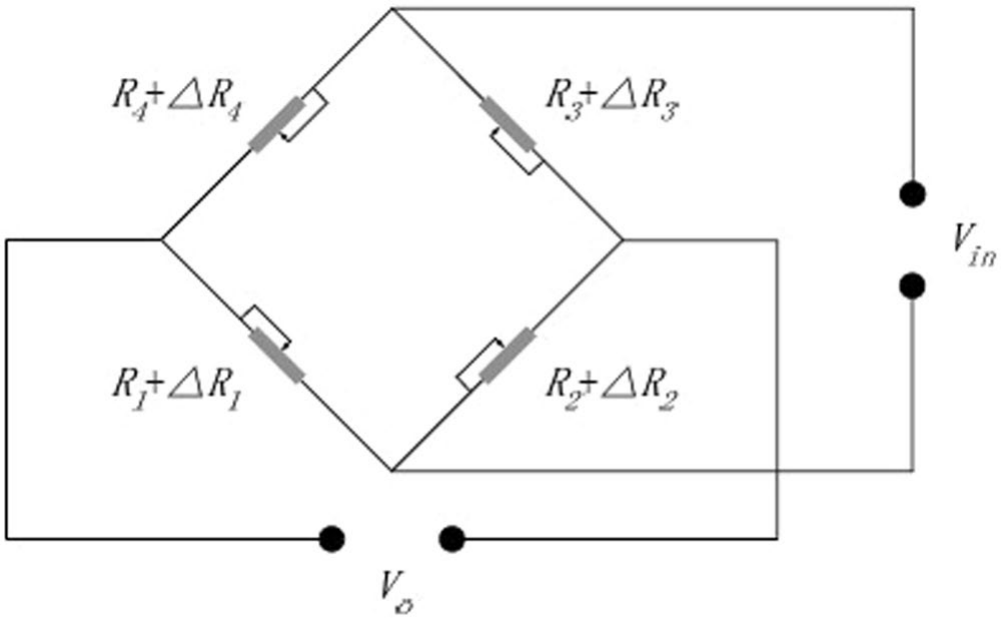
The circuit diagram of the temperature sensor.

The temperature sensor is based on piezoresistive effect using Wheatstone bridge circuit as shown in Fig. 5. The four piezoresistors are divided into two groups, R_1(3)_ and R_2(4)_, respectively. The two piezoresistors, R_1(3)_, are placed along the 〈110〉 crystal direction, while the other two piezoresistors, R_2(4)_, are placed along the 〈1$\overline{1}$0〉 crystal direction. The strain directions of the four piezoresistors are all along the 〈110〉 crystal direction. The relationship between the coefficient of piezoresistance and the direction of current and strain is shown in Table 2. According to Table 2, the piezoresistance coefficient of R_1(3)_ and R_2(4)_ are (*π*_11_ + *π*_12_ + *π*_44_)/2 and (*π*_11_ + *π*_12_ − *π*_44_)/2 respectively. When the square resistance of p-type piezoresistors is in the range of 100–450 Ω/▯, the *π*_11_ and *π*_12_ coefficient are much less than *π*_44_ ^13^. Because the block resistances of piezoresistors in this paper are 100 Ω/▯ and 400 Ω/▯, the piezoresistance coefficient of R_1(3)_ andR_2(4)_ can be expressed as *π*_44_/2 and -*π*_44_/2 respectively.

**Table 2:** The relationship between the coefficient of piezoresistance and the direction of current and strain.

Direction of strain	Direction of current	Coefficient of piezoresistance
〈100〉	〈100〉	*π*_11_
〈100〉	〈010〉	*π*_12_
〈110〉	〈110〉	(*π*_11_ + *π*_12_ + *π*_44_)/2
〈110〉	〈1$\overline{1}$0〉	(*π*_11_ + *π*_12_ − *π*_44_)/2

Under the effect of stress *σ*, variations of piezoresistances are expressed as:

23 $${\rm{\Delta }}{R}_{1}={\rm{\Delta }}{R}_{3}=-\,{\rm{\Delta }}{R}_{2}=-\,{\rm{\Delta }}{R}_{4}=R\frac{{\pi }_{44}}{2}\sigma $$

When the input voltage of the sensor is *V*_*i*_*n*, the output voltage *V*_*o*_ can be calculated as:

24 $${V}_{o}={V}_{in}(\frac{{R}_{1}+{\rm{\Delta }}{R}_{1}}{{R}_{1}+{\rm{\Delta }}{R}_{1}+{R}_{4}+{\rm{\Delta }}{R}_{4}})-{V}_{in}(\frac{{R}_{2}+{\rm{\Delta }}{R}_{2}}{{R}_{2}+{\rm{\Delta }}{R}_{2}+{R}_{3}+{\rm{\Delta }}{R}_{3}})={V}_{in}\frac{{\rm{\Delta }}R}{R}={V}_{in}\frac{{\pi }_{44}}{2}\sigma $$

Based on equations (22) and (24), the output voltage *V*_*o*_ can be expressed as:

25 $${V}_{o}={V}_{in}\frac{{\rm{\Delta }}R}{R}={V}_{in}\frac{{\pi }_{44}{\sigma }_{4max1}}{2}={V}_{in}\frac{{\pi }_{44}{\rm{\Delta }}T}{2}(\frac{{K}_{4}}{{t}_{4}}+\frac{J{E}_{4}{t}_{4}}{2})$$

where Δ*R* is the change of piezoresistance due to deformation. The values of *J* and *K*_4_ are just dependent on the Young’s modulus *E*_*i*_, the thermal expansion coefficient *α*_*i*_ and thickness *t*_*i*_ (*i* = 1, 2, 3, 4), and independent of the length *L* and the width *b*. As a result, the output voltage of the Wheatstone bridge is directly proportional to the temperature change, and independent of the length and width of the cantilever.

**Figure 6:**
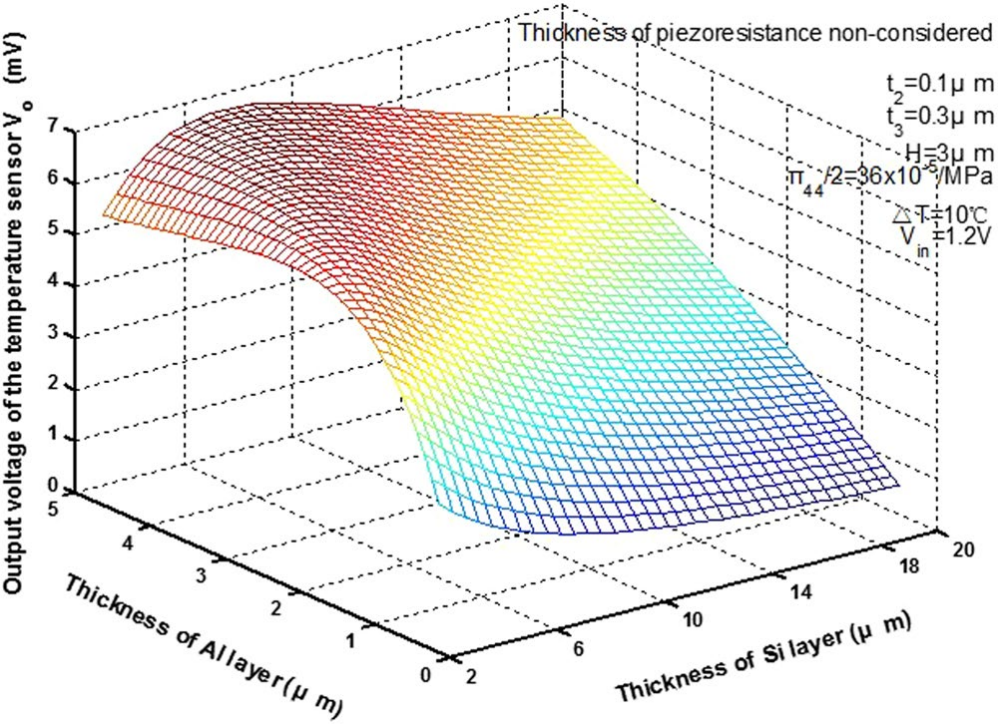
Output voltage versus the thickness of Al and Si, when thickness of pizoresistors is not considered.

Figure 6 shows the output voltage *V*_*o*_ of the temperature sensor as a function of the thickness of the Al layer, *t*_1_, and the thickness of the Si layer, *t*_4_, under the conditions of temperature change Δ*T* = 10 °C, the thickness of Si_3_N_4_ layer *t*_2_ = 0.1 *μ*m, the thickness of S*i*O_2_ layer *t*_3_ = 0.3 *μ*m, input voltage *V*_*in*_ = 1.2 V, the thickness of the Al layer, *t*_1_, in the range 0.5 *μ*m–5 *μ*m, the thickness of the Si layer, *t*_4_, in the range 3 *μ*m–20 *μ*m, and the thickness of pizoresistance is not considered.

The curve shows the output voltage *V*_*o*_ increases rapidly when the thickness of the Al layer, *t*_1_, increases and thickness of the Si layer, *t*_4_, is in the range 6 *μm*–20 *μm*. On the contrary, the output voltage *V*_*o*_ decreases slowly when the thickness of the Si layer, *t*_4_, increases and the thickness of the Al layer, *t*_1_, is in the range 0.5 *μm*–3 *μm*. The thickness of material is very important to the output characteristic of the piezoresistive sensor. According to this figure and the process conditions, the thickness of material can be selected to realize the temperature sensor with high sensitivity.

## Simulation of the Temperature Sensor

**Figure 7:**
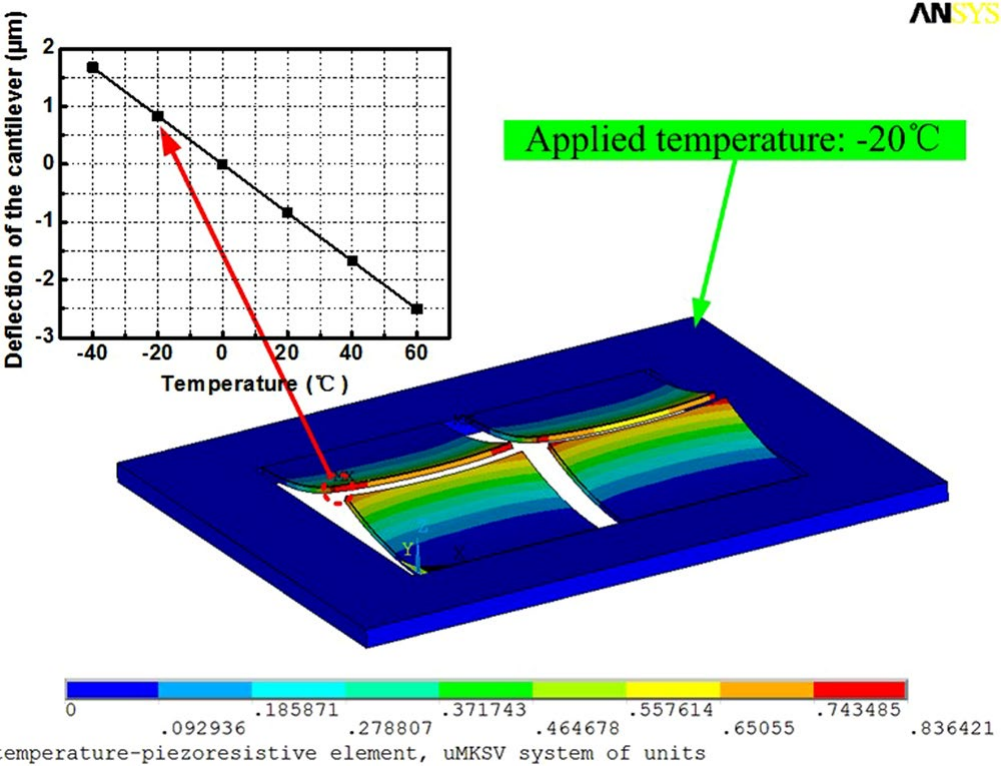
Deflection distribution of the multi-layer cantilevers (400 *μm* long, 500 *μm* wide and 16 *μm* thick) under steady-state conditions.

The sensor is designed and analyzed by the finite element software ANSYS. As shown in Fig. 7, deflection distribution of the sensor’s multi-layer cantilever (400 *μ*m long, 500 *μ*m wide and 16 *μ*m thick) is given by ANSYS, when the applied temperature is −20 °C.

**Figure 8:**
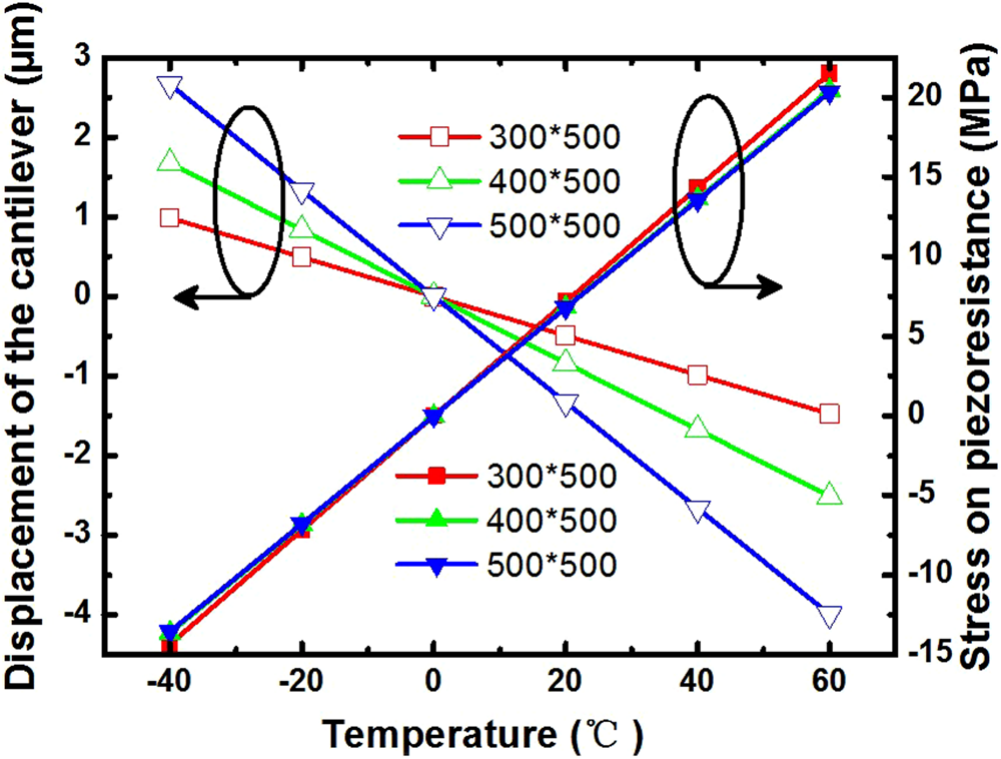
Stress on the piezoresistor and displacement of the temperature sensor cantilevers versus the temperature (The sensors have the same width and different lengths).

Since the coefficient of thermal expansion of the Al layer is larger than that of the Si layer, the cantilevers’ maximum vector displacement occurs at the top of the cantilever and decreases when the temperature rises from −40 °C to 60 °C (Fig. 8). The maximum stress applied on the piezoresistors occurs on the surface of the piezoresistors and increases when the temperature rises from −40 °C to 60 °C (Fig. 8). The cantilevers’ maximum vector displacement is significantly affected by the cantilevers’ length, but the maximum stress applied on the piezoresistors is nearly independent of the cantilevers’ length. The sensor’s sensitivity is closely related to the maximum stress while it has nothing to do with the cantilevers’ length. As a result, the size of the sensor can be reduced, while keeping the sensor’ sensitivity nearly constant.

## Results and Discussion

**Figure 9:**
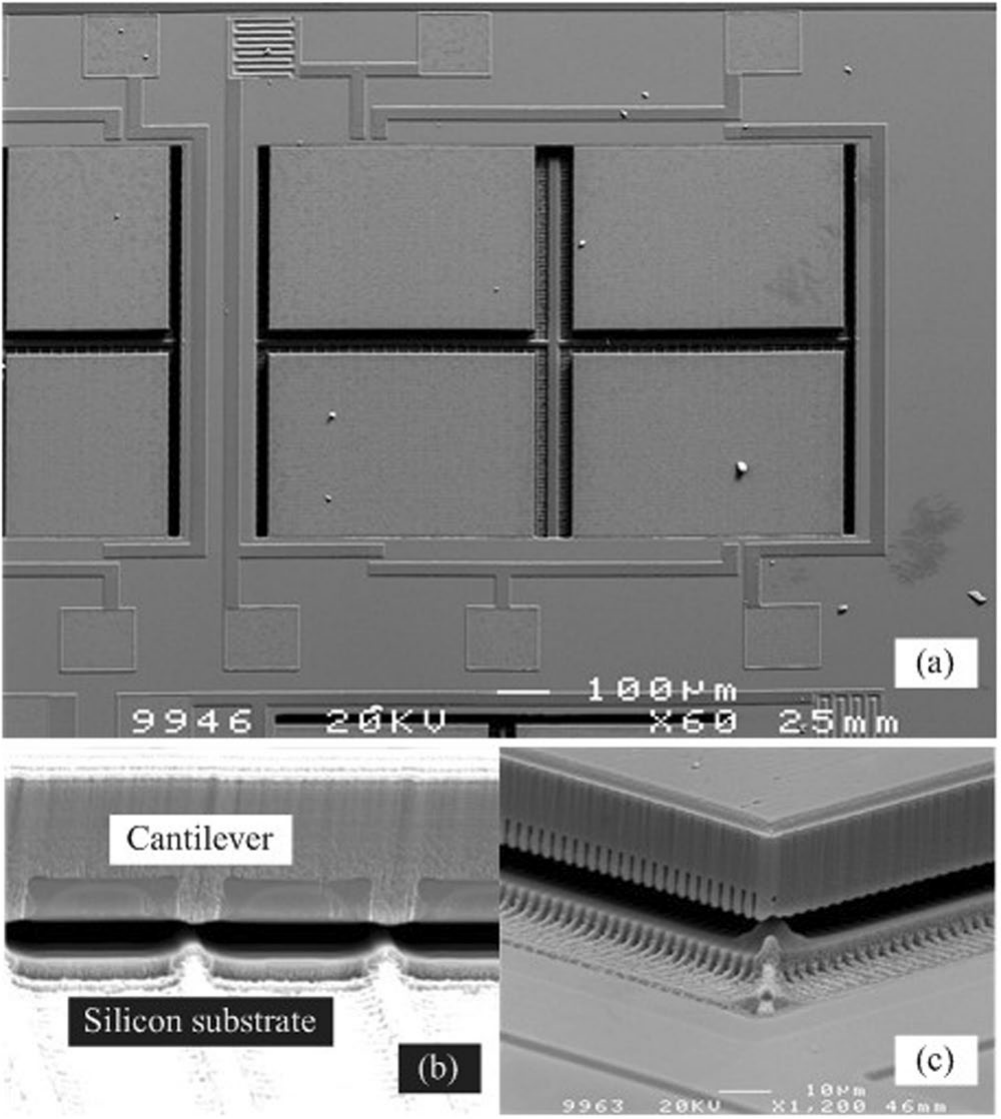
SEM images of the temperature sensor. (**a**) The sensor connected as a Wheatstone bridge. (**b**,**c**) The gap between the bottom of cantilevers and the silicon substrate.

Since the monocrystalline Si micro-structures have excellent mechanical properties, the cantilevers do not subside or adhere (Fig. 9). The temperature sensor contains four piezoresistive elements, which are connected together according to the Wheastone bridge. The sensors were wire-bonded and measured.

**Figure 10:**
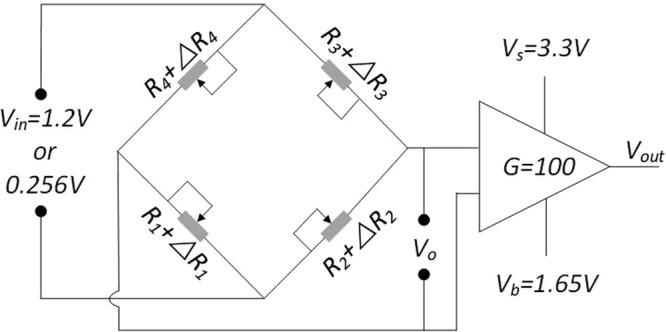
Schematic of temperature sensor test circuit.

As shown in Fig. 10, the temperature sensors are characterized by applying a commonly used circuit for Wheatstone bridges. The input voltage *V*_*in*_ is 1.2 *V* or 0.256 *V*. The gain G and the bias voltage *V*_*b*_ of the amplifier are 100 and 1.65 *V* respectively. In order to compare with the model calculation and finite element simulation, the output voltage *V*_0_ should be obtained by the equation:

26 $${V}_{o}=({V}_{out}-{V}_{b})/G$$

**Figure 11:**
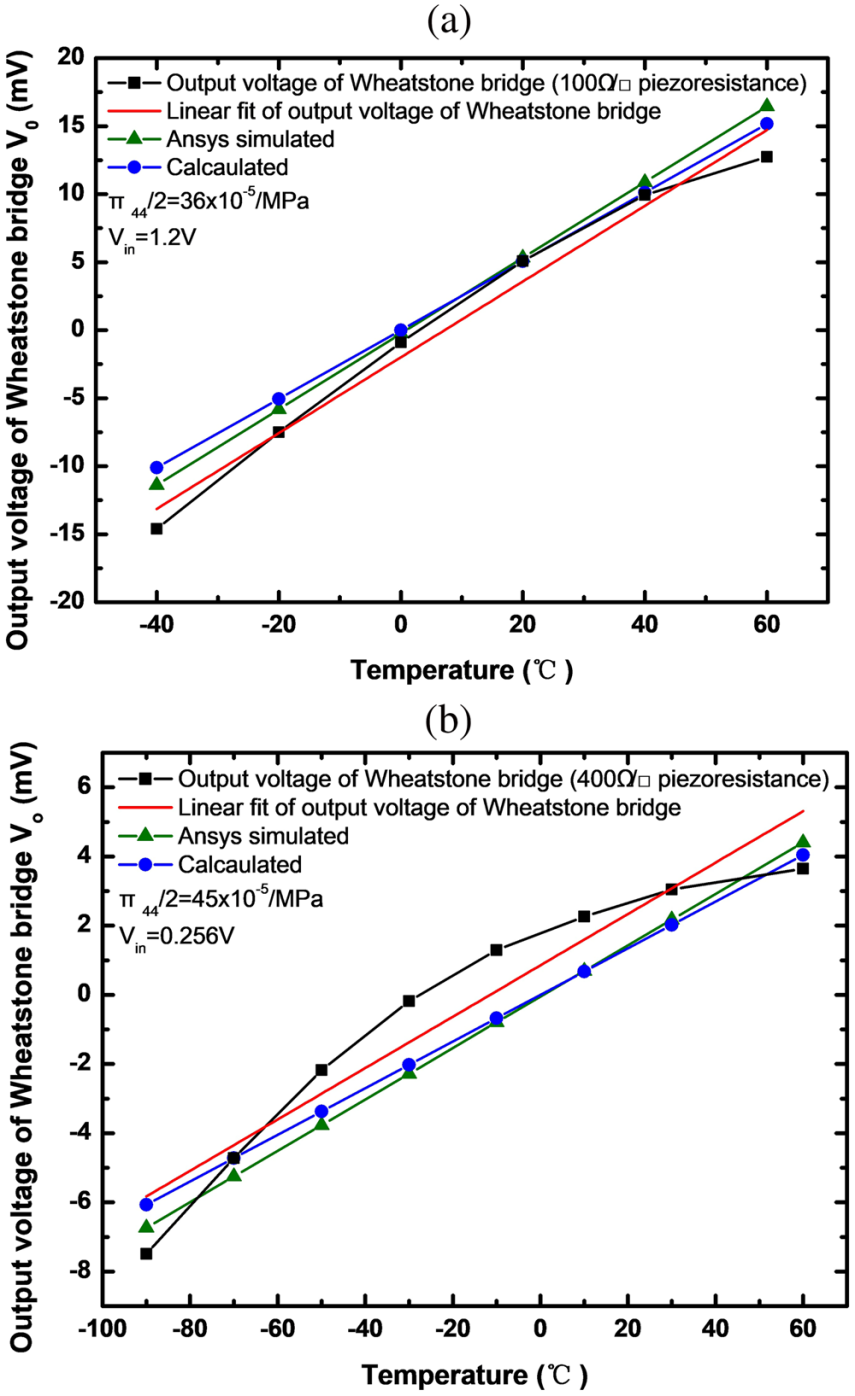
Output voltage of the temperature sensors versus the temperature (**a**) The block resistance of piezoresistance is 100 Ω/▯. (**b**) The block resistance of piezoresistance is 400 Ω/▯.

The diagram in Fig. 11 shows the output voltage of the temperature sensors as a function of temperature. Both the diagrams show 4 curves: the blue curve represents the theoretical calculation results; the green curve represents the finite element simulation results; the black curve represents the measured results and the red curve is the linear fitting curve of the measured results. The sensitivities of the sensors are obtained by the linear fitting. Figure 11(a) shows the output voltage of the temperature sensor with 100 Ω/▯ piezoresistance. The sensitivities obtained based on the measured results, theoretical calculation and finite element simulation are 0.2785 mV/°C,0.253 mV/°C and 0.2735 mV/°C respectively. Figure 11(b) shows the output of the temperature sensor with 400 Ω/▯ piezoresistance and the sensitivities obtained based on the measured results, theoretical calculation and finite element simulation are 0.0743 mV/°C, 0.0675 mV/°C and 0.0742 mV/°C respectively. The theoretical calculation and finite element simulation are close to the measured results. The finite element simulation is in good agreement with the measured results. The difference between the theoretical calculation and the measured results is greater than that between the finite element simulation and the measured results. The reason for this difference is that the boundary conditions of the multi-layer cantilever and the temperature coefficient of the material parameters are ignored in the process of establishing the theoretical model.

By amplifying the output voltage of the wheatstone bridge, the sensitivities of the sensors are 27.9 mV/°C with 100 Ω/▯ piezoresistance between −40 °C to 60 °C and 7.4 mV/°C with 400 Ω/▯ piezoresistance between −90 °C to 60 °C.

The non-linearity of the sensors is obtained by the linear fitting, which uses the least square method. The non-linearity represents deviation of the experimental data from the linear fit. The non-linearity of the temperature sensor with 100 Ω/▯ piezoresistance is 1.67%, which is less than the non-linearity (8.54%) of the sensor with 400 Ω/▯ piezoresistance. The reason for the difference of the nonlinearity of the two sensors is that the temperature dependence of the *π*_44_ coefficient decreases with increasing impurity concentration^12^ and the impurity concentration of 100 Ω/▯ piezoresistance is greater than that of 400 Ω/▯ piezoresistance.

Based on equation (25), the piezoresistance’s relative change rate of the temperature sensor be calculated as:

27 $$d(\frac{{\rm{\Delta }}R}{R})/dT=d(\frac{{V}_{o}}{{V}_{in}})/dT$$

The piezoresistance’s relative change rate is 0.0232%/°C with 100 Ω/▯ piezoresistance and 0.029%/°C with 400 Ω/▯ piezoresistance by calculation. The ratio of the two piezoresistance’s relative change rate is 0.8, which is consistent with the ratio of two piezoresistance’s *π*_44_ coefficient.

Since the temperature sensor proposed in this paper has not been encapsulated, its time constant has not been measured. Since the size of the piezoresistive temperature sensor proposed in this paper is similar to the size of the capacitive temperature sensor proposed by Dr. Ma, the two sensors have similar heat capacities and response time. The measured results show that the response time of the sensor proposed by Dr. Ma is less than 40 ms after encapsulation^14^. As a result, the piezoresistive temperature sensor proposed in this paper also has a low response time theoretically.

## Conclusion

A modified miniature monocrystalline silicon piezoresistive temperature sensor is presented in this paper. It is fabricated by a commercial surface micromachining CMOS MEMS process (the 6 inch CMOS MEMS process - CSMC Technologies). An effective way is proposed to reduce the size of the sensor, while keeping the sensor’ sensitivity nearly constant. Measurement results demonstrate that the temperature sensors can operate in the range of −90 °C to 60 °C, while traditional IC PN junction temperature sensor doesn’t work in this range. The sensor has the advantages of small size and low heat capacity, which make its response time shorter than that of the traditional Pt resistance temperature sensor.

In a word, temperature sensor proposed in this paper can be used in radiosondes for its low operating temperature (as low as −90 °C), small size (below 1 mm^2^) and low heat capacity.

## Methods

In this paper, the proposed temperature sensor is achieved on the epitaxial Si substrate, which had been used in CMOS IC from around 20 years ago^15,16^. The deficiency between the process used in this work and the traditional epitaxial silicon CMOS process is that the process we proposed is a pre-CMOS MEMS process, which has a cavity with around 5 *μ*m depth on the Si substrate. It will not affect the CMOS IC process.

**Figure 12:**
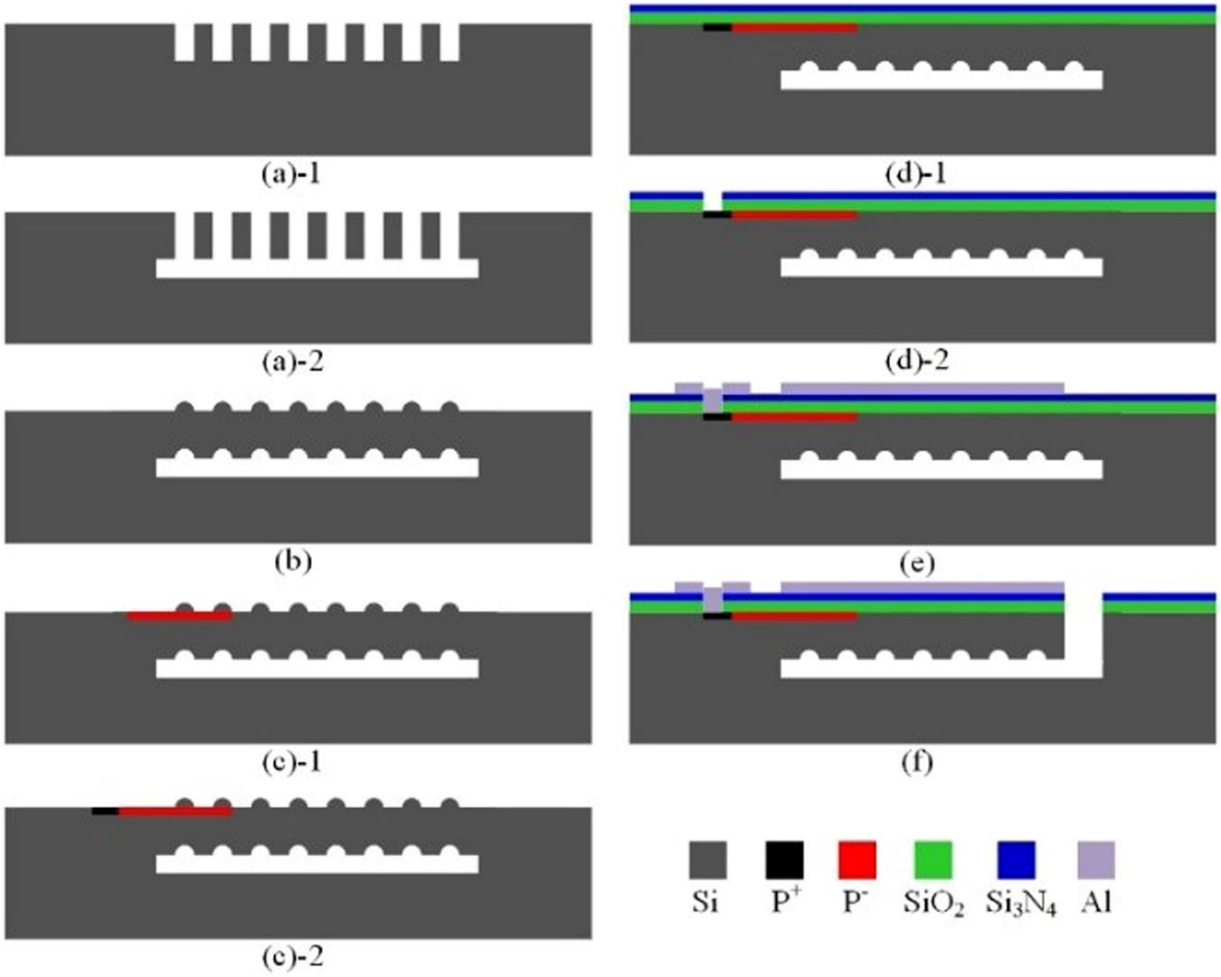
The fabrication of the piezoresistive temperature sensor.

Figure 12 shows that the miniature temperature sensors were fabricated by a surface silicon micromachining CMOS MEMS process^17^. Firstly, the shallow grooves with 5 *μ*m and the cavity with 5 *μ*m were achieved on the monocrystalline Si substrate by reactive ion etching (RIE) process (Fig. 12(a)-1,2). Secondly, the epitaxial Si with 13 *μ*m was deposited on the Si substrate by low pressure chemical vapor deposition (LPCVD) process (Fig. 12(b)). Thirdly, the piezoresistor area and the heavily implanted area were formed by ion injection process with different degrees (Fig. 12(c)-1,2). Fourthly, Si_3_N_4_/SiO_2_ was deposited on the epitaxial Si by LPCVD process and the contact holes were formed by lithography process and etching process (Fig. 12(d)-1,2). Fifthly, the Al film with 2 *μ*m was sputtered and etched on the Si wafer (Fig. 12(e)). Finally, the monocrystalline silicon micro-structures were achieved by deep RIE process (Fig. 12(f)).
